# Effect of Depth-Dependent Integrated Visual Field on Vision-Related Quality of Life in Glaucoma

**DOI:** 10.1167/tvst.13.10.27

**Published:** 2024-10-17

**Authors:** Mehrdad Gazanchian, Nomdo M. Jansonius

**Affiliations:** 1 Department of Ophthalmology, University of Groningen, University Medical Center Groningen, Groningen, The Netherlands; 2 Graduate School of Medical Sciences (Research School of Behavioural and Cognitive Neurosciences), University of Groningen, Groningen, The Netherlands. e-mail: n.m.jansonius@umcg.nl

We read with interest the article by Liu et al.^1^ titled “A Depth-Dependent Integrated VF Simulation for Analysis and Visualization of Glaucomatous VF Defects.” The integrated visual field (IVF) is a technique to estimate the binocular visual field (VF) from monocular VFs by taking the better sensitivity of each eye in each test location of the VF. Liu et al.^1^ introduced an extension of the IVF concept, the depth-dependent IVF (DD-IVF), by incorporating the influence of fixating at different distances on the IVF. They demonstrated the feasibility of this method by using glaucomatous visual field archetypes with dichotomized pointwise sensitivities (dB values) to simulate DD-IVFs.

It is an intriguing but yet unanswered question if this method contributes to understanding the visual performance of patients with glaucoma in real life. More specifically, it would be interesting to know the impact of “volume scotomas” on vision-related quality of life (VR-QoL) in patients with glaucoma. Volume scotomas are physical volumes from which objects cast their images onto areas of the retina with a reduced sensitivity.^2^ These scotomas may affect functional vision, even if standard binocular perimetry appears normal. For example, in bitemporal hemianopia, although the binocular visual field is normal, there is a volume scotoma behind the fixation point, and this scotoma may cause problems in near activities and depth perception. Mutatis mutandis, the predominantly nasal visual field defects in glaucoma could result in volume scotomas in front of the fixation point.

**Figure 1.**
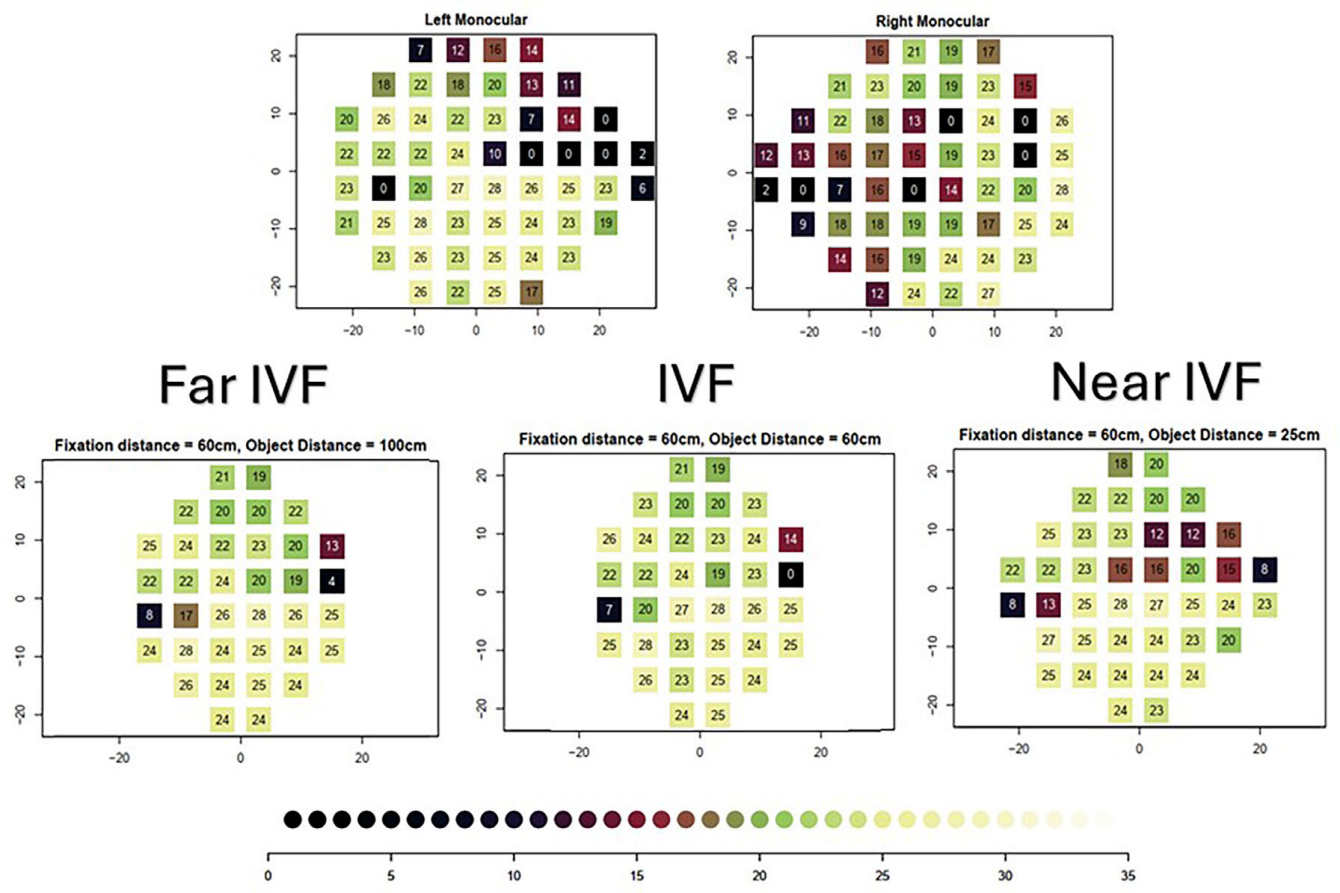
Example of the monocular visual fields, IVF, and near and far IVF in a patient with glaucoma. Axes show eccentricity in degrees; numbers show raw pointwise sensitivities in dB. Figure was created using the “binovisualfield” R package.^1^

We used a data set from a recent study to calculate the DD-IVF and determine its association with VR-QoL.^3^ We included 173 patients with bilateral glaucoma in this analysis (median [interquartile range] mean deviation [MD] was −6.7 [−11.1 to −3.9] dB for the better eye and −14.0 [−19.0 to −10.0] dB for the worse eye). Patients had completed four questionnaires, including the National Eye Institute Visual Function Questionnaire (NEI-VFQ-25),^4^ the NEI-VFQ-25 Neuro-Ophthalmology Supplement,^5^ the Glaucoma Quality of Life–15 questionnaire,^6^ and a luminance-specific questionnaire developed by Bierings et al.^7^ Following Liu et al.,^1^ we calculated “far IVF” as the IVF when a subject is fixating at 60 cm but the object is at 100 cm and “near IVF” as the IVF when a subject is fixating at 60 cm but the object is at 25 cm (see Fig. 1 for an example). For the calculation of IVF, near IVF, and far IVF, the raw VF sensitivity values were used rather than total deviation plot values. Furthermore, we trimmed the IVF visual field to include only those test locations that appeared in both near and far IVF. We first calculated the (1) pointwise difference and (2) absolute difference between near and far IVF in all VF locations that had both near and far IVF. We then used the mean of the pointwise differences and absolute differences as measures of the effect of seeing in depths other than at the fixation plane. Ordinal multiple regression analysis was used to examine the association between various aspects of VR-QoL and these measures. The analyses (we built a separate model for each VR-QoL aspect) were adjusted for age, sex, IVF mean sensitivity (MS), and the better visual acuity of both eyes. The false discovery rate (FDR) method was used to adjust for multiple comparisons.

**Figure 2.**
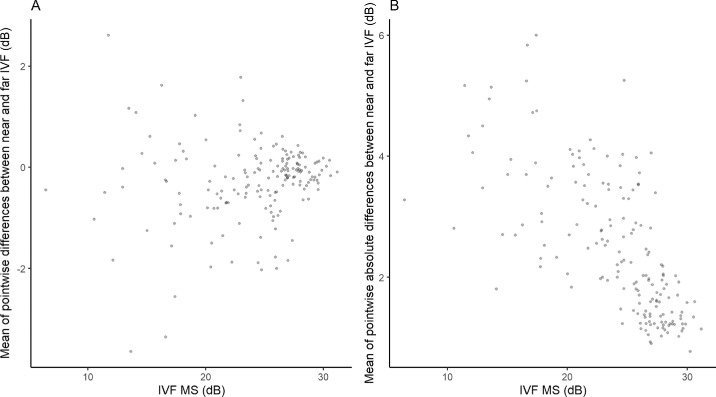
Mean of pointwise differences (**A**) and absolute differences (**B**) between near and far IVF as a function of IVF MS.

**Figure 3.**
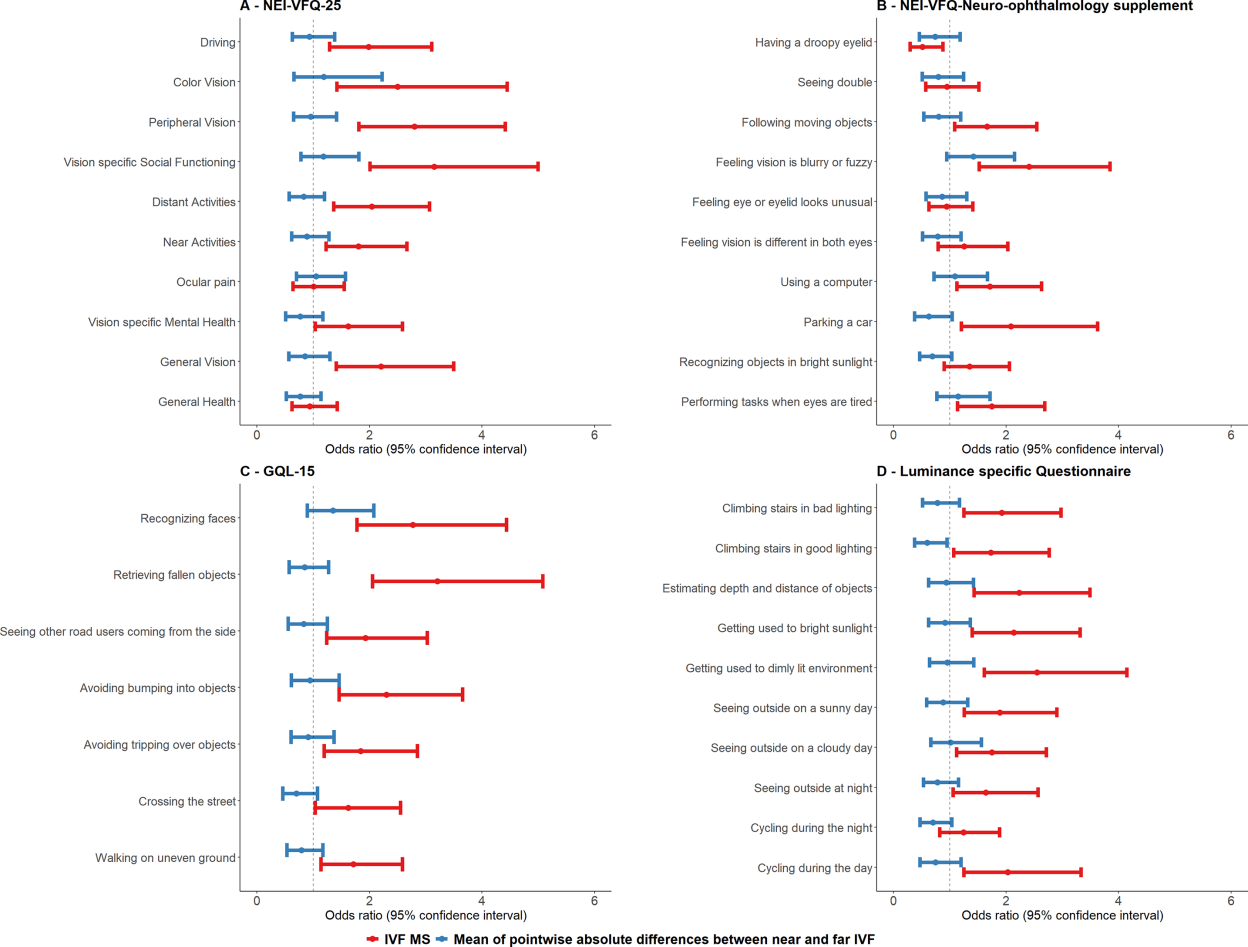
Ordinal multiple regression results for the effect of IVF MS (*blue*) and mean of pointwise absolute differences between near and far IVF (*red*) on VR-QoL as assessed with the (**A**) NEI-VF-25, (**B**) NEI-VF-25 neuro-ophthalmic supplement, (**C**) Glaucoma Quality of Life–15 (GQL-15), and (**D**) a luminance-specific questionnaire, adjusted for age, sex, and better eye visual acuity. Poor VR-QoL corresponds to an odds ratio >1 for IVF and <1 for the difference between near and far IVF.

Figure 2 shows the mean of the pointwise differences and absolute differences between near and far IVF as a function of disease severity (IVF MS); the means of the pointwise differences (in either direction) and the absolute differences tend to become more pronounced with worsening of the disease. Figure 3 presents the effects of the mean of the pointwise absolute differences between near and far IVF on VR-QoL. We did not find any significant association. The only item that approached significance was “climbing the stairs in good lighting” (*P* = 0.029, FDR-adjusted *q* = 0.063). We repeated the analyses using the difference (as opposed to absolute difference) between near and far IVF, and similar results were found (with now borderline significance for “crossing the street” and “feeling vision is fuzzy or blurry”; data not shown). This suggests that volume scotomas, while they may have a negative impact on some aspects of vision in specific cases (like in bitemporal hemianopia), seem not to be a major determinant of VR-QoL in patients with glaucoma. Our results do not rule out, however, that volume scotomas play a role in some patients with glaucoma with rather severe VF defects in certain specific visual tasks.

In conclusion, the article by Liu et al.^1^ presented a valuable technique for estimating volume scotomas. When applying this technique to a large data set of patients with glaucoma, no specific effects of volume scotomas on VR-QoL could be uncovered.
